# To fast or not to fast: Lipid measurement and cardiovascular disease risk estimation in rural sub-Saharan Africa

**DOI:** 10.7189/jogh.10.010407

**Published:** 2020-06

**Authors:** Isabelle T Yang, Linda C Hemphill, June-Ho Kim, Prossy Bibangambah, Ruth Sentongo, Bernard Kakuhire, Jorge Plutzky, Yap Boum, Alexander C Tsai, Samson Okello, Mark J Siedner

**Affiliations:** 1 Geisel School of Medicine at Dartmouth, Hanover, New Hampshire, USA; 2 Harvard Medical School, Boston, Massachusetts, USA; 3 Massachusetts General Hospital, Boston, Massachusetts, USA; 4 Brigham and Women’s Hospital, Boston, Massachusetts, USA; 5 Mbarara University of Science and Technology, Mbarara, Uganda; 6 Epicentre Research Base, Mbarara, Uganda

## Abstract

**Background:**

Cardiovascular disease (CVD) morbidity and mortality are increasing in sub-Saharan Africa (sSA), highlighting the need for tools to enable CVD risk stratification in the region. Although non-HDL-cholesterol (nHDL-C) has been promoted as a method to measure lipids without a requirement for fasting in the USA, its diagnostic validity has not been assessed in sSA. We sought to estimate: 1) the association between LDL-cholesterol (LDL-C) and nHDL-C, 2) the impact of fasting on their measurement, and 3) their correlation with carotid atherosclerosis, within a rural Ugandan population with high HIV prevalence.

**Methods:**

We collected traditional CVD risk factors, blood for serum lipid levels, self-reported fasting status, and performed carotid ultrasonography in 301 participants in rural Uganda. We fit regression models, stratified by fasting status, to estimate associations between carotid intima media thickness (cIMT), LDL-C, and nHDL-C.

**Results:**

Median age was 50 years (interquartile range = 46-54), 49% were female, 51% were HIV-positive, and at the time of blood collection, 70% had fasted overnight. Mean LDL-C, nHDL-C, and triglycerides in the non-fasting and fasting groups were 85 vs 88 mg/dL (*P* = 0.39), 114 vs 114 mg/dL (*P* = 0.98), and 130 vs 114 mg/dL (*P* = 0.05) mg/dL, respectively. In unadjusted models, mean cIMT (mm) was associated with both increased LDL-C (β = 0.0078 per 10mg/dL, *P* < 0.01) and nHDL-C (β = 0.0075, *P* < 0.01), and these relationships were similar irrespective of fasting status. After adjustment for traditional CVD risk factors, we observed similar associations, albeit with muted effect sizes within the fasting group.

**Conclusions:**

We found a high correlation between LDL-C and nHDL-C, and both were correlated with cIMT, irrespective of fasting or HIV serostatus in rural Uganda. Our findings support use of either fasting or non-fasting serum lipids for CVD risk estimation in rural sSA.

Cardiovascular diseases (CVDs) are a major cause of morbidity worldwide. They accounted for 31% of deaths globally in 2016, 78% of which are estimated to have occurred in low- and middle-income countries [1]. Although the CVD-related death rate has fallen in high-income countries with improved care and risk management [2], mortality from CVD is increasing in low- and middle-income countries [3]. Despite recent efforts, the CVD epidemic has yet to be accurately quantified in these regions, and research on effective screening and treatment for CVD both for public health policy planning and individual management is stated as a major research priority by the World Health Organization, the US National Institutes of Health and other organizations [3-8]. Most studies establishing measurement and validity of CVD risk surrogates were conducted in higher income countries, raising questions about generalizability to other settings and populations. Thus, there is an important need for developing and validating effective techniques for screening and care of CVD in low-income countries.

Long-standing consensus establishes low-density lipoprotein cholesterol (LDL-C) as a strong predictor of future cardiovascular events [9]. However, there is accumulating evidence challenging its selection as the optimal lipid measure for CVD risk estimation and therapeutic targeting [9-12]. The referent method for direct measurement of LDL-C (beta quantitation) requires ultracentrifugation and precipitation of apoprotein-B particles [13], which entails specialized laboratory equipment and is impractical outside of most reference laboratories. In contrast, indirect estimation via the Friedewald equation, which is dependent on accurate estimation of triglycerides (TGs) and TGs values within an acceptable range, requires patients to fast for eight hours prior to phlebotomy [14]. However, pre-collection fasting poses a number of challenges. In rural sub-Saharan Africa (sSA), both patient and health care-system factors, such as long travel distances and long wait times at health centers [15,16], can prevent prolonged fasting. Furthermore, measured LDL-C from either method actually includes LDL along with unpredictable amounts of intermediate-density lipoprotein (IDL) and lipoprotein(a), and is not a true reflection of LDL concentration [14].

More recent studies offer increasing support for the use of non-high-density lipoprotein cholesterol (nHDL-C) as a superior measure for risk CVD assessment and treatment targeting [9-12,17]. nHDL-C includes cholesterol types that have been associated with CVD, including very-low density lipoprotein, IDL, LDL, and lipoprotein(a) [9,14]. In addition, measuring nHDL-C is procedurally simpler, since it only requires measuring total cholesterol and HDL-C, and is not affected by fasting [14]. Studies in Western Europe and North America have demonstrated that nHDL-C is equally if not more predictive of CVD events compared with LDL-C. In these studies, the estimated associations were noted irrespective of fasting status [18,19].

The applicability of these findings to other regions in the world is unclear. Relationships between cholesterol and CVD risk may differ in sSA compared with Western European and North American countries due to differences in genetics, diet, and other environmental characteristics. For example, prior studies of South Asian populations have found elevated risk for acute myocardial infarction at much lower LDL-C levels compared with Caucasian populations or other ethnic groups [20,21]. Possible causes include higher LDL particle burden or elevated apolipoprotein (apo) B [20,22]. These studies offer evidence against extrapolating from treatment guidelines based on Western European or North American standards to other settings globally without further investigation [23]. However, to the best of our knowledge, no study in sSA has examined relationships between LDL-C, nHDL-C, and CVD risk. Unique genetics, behavioral factors, high HIV prevalence and corresponding widespread antiretroviral therapy are all potential modifiers of this relationship. Moreover, practical considerations of lipid assessment, such as cost and availability of testing, as well as cultural appropriateness of fasting, will be critical to planning regional CVD risk assessment strategies [19].

The overarching aim of this analysis was to provide data to inform public health and clinical programs in the region on the optimal means of using lipid testing for CVD risk profiling. We sought to compare the strength of association between carotid intima media thickness (cIMT) [24] and both LDL-C and nHDL-C, and to assess how fasting affects these relationships. A secondary goal of the study was to explore how HIV infection affects CVD risk assessment via lipid profiling. We hypothesized that nHDL-C would be superior to LDL-C, primarily due to diminished correlation between non-fasting LDL-C and cIMT, and that treated HIV infection would not modify the relationships between cholesterol levels and CVD risk.

## MATERIALS AND METHODS

### Study population

Data from this analysis was drawn from the Ugandan Non-communicable Diseases and Aging Cohort (UGANDAC) Study (NCT02445079). The study design is a longitudinal cohort study which aims to describe non-communicable disease morbidity and aging among an older, ambulatory population in rural Uganda. The cohort is comprised of two sub-groups: people over 45 years of age living with HIV (PLWH) on antiretroviral therapy (ART) in outpatient care and an age and sex-matched, HIV-uninfected, and population-based comparator group [25-27]. Participants are seen annually for completion of questionnaires, specimen collection, and cardiovascular diagnostic measurements.

### Data collection

Data used in this analysis were collected at initial enrollment. Participants completed surveys on CVD and risk factors, including smoking history [28], self-reported CVD history, and physical activity level [29]. Participants were requested to fast after midnight on the day of study procedures, and fasting status was self-reported. Resting, bilateral blood pressure was collected using digital sphygmomanometers (Omron 10 Series, Omron Healthcare, Chicago, IL, USA). Body mass index (BMI) was calculated from height and weight measurements. Blood was drawn and centrifuged for serum separation within 2 hours, and stored at -80°C until the time of testing. Lipid testing was done in Boston, Massachusetts at LabCorp using the Abbott Architect Clinical Chemistry Analyzer (Abbott Diagnostics, Abbott Park, IL, USA). Participants were enrolled in two waves. In the first wave (202/204 with serum available), a full lipid panel including direct LDL-C was measured. In the second wave (99/105 with serum available), LDL-C was calculated from total cholesterol, HDL-C and triglycerides using the Friedewald equation.

Carotid ultrasonography for common carotid intima-media thickness (cIMT) measurement was conducted using SonoSite M-Turbo (SonoSite, Bothell, WA, USA) as described previously [30] by one of two study staff members trained in carotid ultrasonography at the University of Wisconsin [31]. This technique has been validated as a noninvasive, sensitive, and reproducible means of estimating carotid atherosclerotic burden [32-35]. Six images of the carotid artery were taken per participant, including images from the anterior, lateral, and posterior angles on both the left and right carotid artery. Images were reviewed by a board-certified cardiologist, and images of inadequate quality were removed from analysis. Among participants included in our analysis, 69 (4%) of the 1806 expected measurements were removed due to poor quality. We used semi-automated border-detection software to estimate common carotid cIMT just proximal to the carotid bulb (SonoCalc, version 5.0; SonoSite) [36]. cIMT measurements were interpreted by two different operators. The two operators independently interpreted 120 images, and an inter-operator analysis was conducted to confirm the comparability of measurements (Pearson correlation *r* = 0.98, *P* = 0.24). Operators were blinded to participant characteristics, including fasting status, at the time of cIMT measurement.

### Statistical analyses

We used standard data summarization, tabulation, and box-plotting methods to summarize cohort characteristics by fasting status. We characterized LDL-C and nHDL-C with mean and standard deviation, and TGs with median and interquartile range based on their distributions. We produced histogram plots for continuous variables to confirm normal or non-normal distributions. We compared characteristics by fasting status using *t* tests for normally distributed, continuous variables, Wilcoxon rank-sum tests for non-normally distributed, continuous variables, and χ^2^ tests for categorical variables. We produced scatter plots to graphically depict relationships between LDL-C and nHDL-C. Correlation was evaluated with Pearson (*r*) coefficients, which were then compared between fasting groups [37]. We then fit linear regression models to estimate associations between each lipid cholesterol and CVD risk both for all participants, and stratified by fasting status. For these models, our dependent variable was subclinical atherosclerosis, as measured by cIMT. Our primary explanatory variables of interest were nHDL-C and LDL-C. Correlation between lipid cholesterol and cIMT was calculated using Spearman correlation (ρ). Finally, we fit adjusted regression models for each lipid measure, adjusting for age [33,38], sex [39], HIV status [40,41], BMI [42], blood pressure [43,44], smoking history [44,45], CVD history [46], and physical activity category [47], as potential confounders of the relationship between lipid measures and cIMT. In sensitivity analyses, we repeated correlation and regression model procedures restricting the sample to instances in which direct LDL-C measurements were available. To further explore the robustness of our findings to potential unobserved confounding, we dichotomized LDL-C into <100 and ≥100mg/dL and nHDL-C into <130 and ≥130mg/dL and fit multivariable regression models specifying cIMT as the dependent variable and adjusting for the same covariates. We calculated e-values for these adjusted regression models to estimate the strength of association required by an unobserved confounder with both high lipid cholesterol and cIMT in order to explain the results [48]. We used Stata/MP 15.0 (Statacorp, College Station, Texas, USA) for all statistical analyses.

### Ethics statement

Study procedures were reviewed and approved by the human subjects committees at the Mbarara University of Science and Technology and Partners Healthcare. Consistent with national guidelines, we also obtained clearance for the study from the Ugandan National Council of Science and Technology. The study protocol follows the guidelines as described in the 1975 Declaration of Helsinki. All participants signed written informed consent.

## RESULTS

Out of 309 participants enrolled in the UGANDAC study, 301 (97%) completed both blood collection for lipids and carotid ultrasound, and were included in the study. Five were excluded due to inadequate cIMT measurements, and three were excluded due to missing lipid data. Among the 301 total participants, 202 participants underwent further lipid testing for direct LDL-C (83% fasting). Of the participants included in the study, 212 (70%) reported an overnight fast. **Table 1** summarizes CVD risk factors by fasting status. 94% of participants living with HIV were virologically suppressed, the majority (80%) were on AZT/3TC/NVP or AZT/3TC/EFV, and only 7% were on protease inhibitors. The fasting subgroup was slightly younger (median age 50 vs 52 years; *P* = 0.009), was more likely to be HIV-positive (56% vs 40%; *P* = 0.016), and had a non-significantly lower mean cIMT (0.666 vs 0.689mm; *P* = 0.0649).

**Table 1:** Cohort characteristics

Characteristic	Total (n = 301)	Non-fasting subgroup (n = 89; 30%)	Fasting subgroup (n = 212, 70%)	*P*-value*
Age (median, IQR)	50 (46-54)	52 (49-54)	50 (46-54)	0.0086
Female sex (n, %)	147 (49)	45 (51)	102 (48)	0.698
HIV-positive (n, %)	154 (51)	36 (40)	118 (56)	0.016
BMI (n, %):				0.132
-BMI cat <18	31 (10)	10 (11)	21 (10)	
-BMI cat 18-25	194 (65)	51 (57)	143 (68)	
-BMI cat 25-30	49 (16)	15 (17)	34 (16)	
-BMI cat >30	27 (9)	13 (15)	14 (7)	
High blood pressure†, (n, %)	47 (16)	17 (19)	30 (14)	0.359
Smoking (n, %):				0.668
-Never	177 (59)	55 (62)	122 (58)	
-Former	90 (30)	26 (29)	64 (30)	
-Current	34 (11)	8 (9)	26 (12)	
History of CVD (n, %)	57 (19)	17 (19)	40 (19)	0.962
Physical activity‡ (n, %):				0.400
-Low physical activity	16 (5)	7 (8)	9 (4)	
-Moderate physical activity	41 (14)	13 (15)	28 (13)	
-High physical activity	244 (81)	69 (77)	175 (83)	
HDL cholesterol level (mg/dL) (mean, SD)	45.99 (13.53)	45.67 (13.17)	46.12 (13.71)	0.794
LDL cholesterol level§ (mg/dL) (mean, SD)	87.18 (30.99)	84.80 (30.07)	88.18 (31.37)	0.389
TG level (mg/dL) (median, IQR)	117 (92-160)	130 (95-178)	114 (91.5-153.5)	0.052
Non-HDL cholesterol level (mg/dL) (mean, SD)	114.22 (34.25)	114.13 (34.50)	114.25 (34.22)	0.979
Total cholesterol level (mg/dL) (mean, SD)	160.21 (36.04)	159.81 (33.96)	160.37 (36.95)	0.902

IQR – interquartile range, BMI – body mass index, CVD – cardiovascular disease, HDL – high-density lipoprotein, LDL – low-density lipoprotein, TG – triglycerides, SD – standard deviation

*P-values comparing participants in the fasting and non-fasting sub-groups were calculated using χ^2^ testing for categorical variables, *t* tests for normally distributed continuous variables, and log-rank testing for non-normally distributed continuous variables.

†High blood pressure defined as systolic blood pressure >140 or diastolic blood pressure >90 mmHg.

‡Physical activity categories were determined by the International Physical Activity Questionnaire (IPAQ) scoring protocol [49].

§LDL cholesterol were calculated using the Friedewald equation [50].

**Figure 1:**
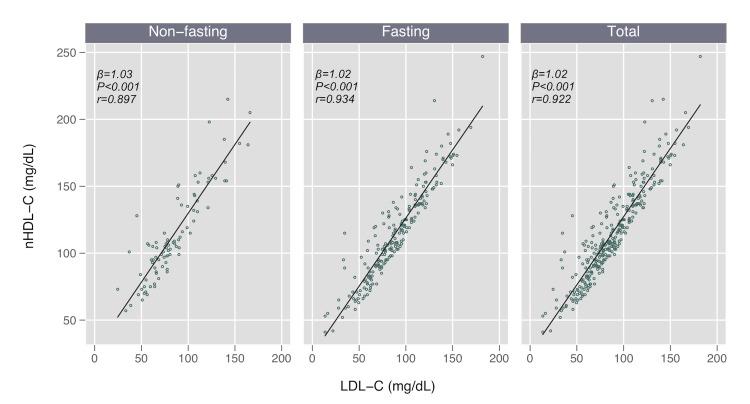
Scatter plots and linear fit lines comparing non-high-density lipoprotein cholesterol (non-HDL-C) and low-density lipoprotein cholesterol (LDL-C), by fasting status.

We found no difference in LDL-C or nHDL-C concentrations by fasting status (*P* > 0.3 for both comparisons), but nominally higher TG levels in the non-fasting subgroup (median 130 vs 114 mg/dL, *P* = 0.100), as expected. We found strong linear relationships between nHDL-C and LDL-C in the total cohort (Pearson *r* = 0.922), and in both fasting (*r* = 0.934) and non-fasting subgroups (*r* = 0.897) (**Table 2**; **Figure 1**). These estimates were similar when restricting the sample to the 202 participants (83% fasting) in which direct LDL-C measurements were available (Pearson *r* = 0.935 total, *r* = 0.962 non-fasting, *r* = 0.931 fasting).

**Table 2:** Non-HDL-C vs LDL-C, by fasting group

	β-value (95% CI)	*P-*value	*r* coefficient (95% CI)
Non-fasting	1.03 (0.920, 1.137)	<0.001	0.897 (0.846, 0.931)
Fasting	1.02 (0.965, 1.072)	<0.001	0.934 (0.914, 0.949)
Total	1.02 (0.970, 1.068)	<0.001	0.922 (0.903, 0.937)

Non-HDL-C – non-high-density lipoprotein cholesterol, LDL-C – low-density lipoprotein cholesterol, CI – confidence interval

*P* = 0.070 comparing non-fasting and fasting *r*-values

**Figure 2:**
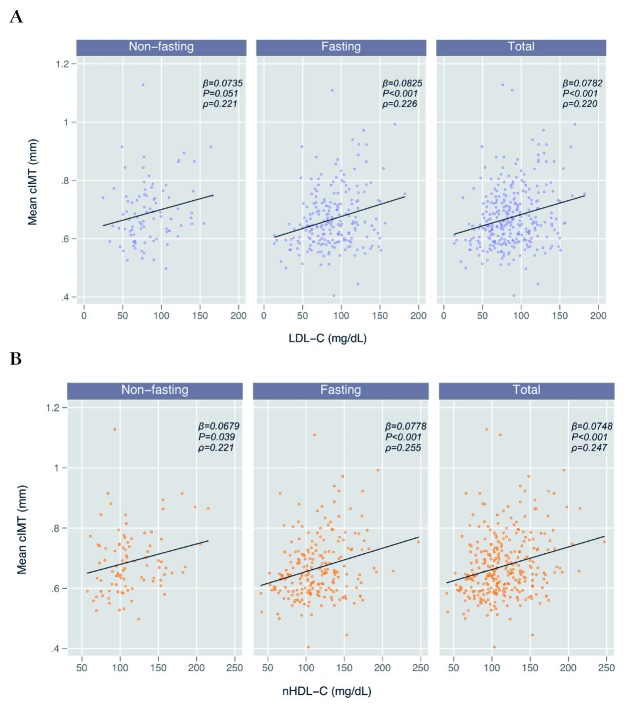
Unadjusted scatter plots and linear fit lines comparing low-density lipoprotein cholesterol (LDL-C) (**A**), non-high-density lipoprotein cholesterol (non-HDL-C) (**B**) and common carotid intima media thickness (cIMT), by fasting status. **Panel A.** Mean cIMT vs LDL-C by fasting status. **Panel B.** Mean cIMT vs non-HDL-C by fasting status. *Reported β-values are per 100mg/dL increase in the corresponding lipid cholesterol level.

In unadjusted regression analyses, mean cIMT was associated with LDL-C in the total cohort (β = 0.0782 per each 100mg/dL increase; *P* < 0.001) as well as in both non-fasting (β = 0.0735; *P* = 0.051) and fasting sub-groups (β = 0.0825; *P* < 0.001) (**Table 3**; **Figure 2**). In a sensitivity analysis among the 202 participants with direct LDL-C measurements, we found similar results, though the association between cIMT and direct LDL-C was insignificant in the non-fasting subgroup (β = 0.0990; *P* = 0.129) (Table S1 in the **Online Supplementary Document**). In our adjusted model, including traditional CVD risk factors, this association remained statistically significant for the entire cohort (β = 0.0501; *P* = 0.003) and in the non-fasting (β = 0.0795; *P* = 0.043), although it was muted in the fasting subgroup (β = 0.0351; *P* = 0.080) (**Table 3**). We found no evidence of effect modification in the relationship between LDL-C and cIMT by fasting status (interaction term *P* = 0.372) or HIV serostatus (interaction term *P* = 0.421) (Table S2 in the **Online Supplementary Document**).

**Table 3:** Unadjusted and adjusted models for regression of lipid types on carotid intima media thickness (mm)

Lipid measure	Fasting group	Unadjusted			Adjusted*			Adjusted† removed age		
**β coefficient (95% CI)**	***P-*value**	**R-squared value**	**β coefficient (95% CI)**	***P-*value**	**R-squared value**	**β coefficient (95% CI)**	***P-*value**	**R-squared value**
**LDL-C level (mg/dL), per 100 unit increase**	Non-fasting	0.0735 (-0.0004, 0.147)	0.051	0.043	0.0795 (0.00267, 0.156)	0.043	0.356	0.0849 (0.00435, 0.165)	0.039	0.282
	Fasting	0.0825 (0.0398, 0.125)	<0.001	0.064	0.0351 (-0.00417, 0.0744)	0.080	0.380	0.0620 (0.0175, 0.106)	0.007	0.177
	Total	0.0782 (0.0411, 0.115)	<0.001	0.055	0.0501 (0.0167, 0.0834)	0.003	0.341	0.0652 (0.0279, 0.102)	0.001	0.167
**Non-HDL-C level (mg/dL), per 100 unit increase**	Non-fasting	0.0679 (0.00364, 0.132)	0.039	0.048	0.0827 (0.0142, 0.151)	0.019	0.369	0.0868 (0.0150, 0.159)	0.018	0.294
	Fasting	0.0778 (0.0387, 0.117)	<0.001	0.068	0.0256 (-0.0116, 0.0628)	0.177	0.376	0.0558 (0.0141, 0.0976)	0.009	0.175
	Total	0.0748 (0.0414, 0.108)	<0.001	0.061	0.0418 (0.0106, 0.0730)	0.009	0.337	0.0592 (0.0245, 0.0938)	0.001	0.165
**Triglycerides level (mg/dL), per 100 unit increase**	Non-fasting	0.0122 (-0.0174, 0.0418)	0.414	0.008	0.0150 (-0.0135, 0.0434)	0.298	0.330	0.0148 (-0.0151, 0.0447)	0.328	0.250
	Fasting	0.0132 (-0.00936, 0.0357)	0.251	0.006	-0.00952 (-0.0311, 0.0120)	0.385	0.373	0.00234 (-0.0225, 0.0272)	0.853	0.146
	Total	0.0144 (-0.00330, 0.0320)	0.111	0.009	-0.00220 (-0.0183, 0.0139)	0.788	0.321	0.00385 (-0.0143, 0.0220)	0.676	0.133

Non-HDL-C – non-high-density lipoprotein cholesterol, LDL-C – low-density lipoprotein cholesterol, CI – confidence interval

*Models adjusted for age, sex, HIV-status, BMI, blood pressure, smoking, history of CVD, physical activity.

†Models adjusted for sex, HIV-status, BMI, blood pressure, smoking, history of CVD, physical activity.

Mean cIMT was also positively and significantly associated with nHDL-C (β = 0.0748; *P* < 0.001) in both non-fasting (β = 0.0679; *P* = 0.039) and fasting (β = 0.0778; *P* < 0.001) subgroups (**Table 3**; **Figure 2**). As with the LDL-C models, this association remained statistically significant after adjustment for other traditional CVD risk factors for the entire cohort (β = 0.0418; *P* = 0.009) and non-fasting (β = 0.0827; *P* = 0.019) subgroup, but was muted within the fasting subgroup (β = 0.0256; *P* = 0.177) (**Table 3**). We again found no effect modification in the relationship between nHDL-C and cIMT by fasting status (interaction term *P* = 0.427) or HIV serostatus (*P* = 0.792) (Table S2 in the **Online Supplementary Document**). Using an approach where we removed individual factors stepwise from the adjusted model, we identified age as the likely confounding factor that explained the difference in estimates between the adjusted and unadjusted fasting models for both LDL-C and nHDL-C (**Table 3**). To lend further support to the possibility of age as a confounder, we analyzed the relationship between lipids and age, stratified by fasting status, and found that age was significantly correlated with LDL-C and nHDL-C in the fasting group (*P* = 0.012 and 0.001, respectively), but not in the non-fasting group *(P =* 0.697 and 0.660) (Table S3 in the **Online Supplementary Document**).

The potential for confounding from unobserved variables was explored via e-value analysis. In adjusted models, with age removed, in which we dichotomized LDL-C and nHDL-C with thresholds of 100mg/dL and 130mg/dL respectively, cIMT was significantly associated with either an LDL-C of 100mg/dL or greater (β = 0.0265; *P* = 0.035) or an nHDL-C of 130mg/dL or greater (β = 0.0326; *P* = 0.010). Using an approximation converting continuous outcomes into risk ratios, we calculated e-value = *RR* + *sqrt* (*RR* × (*RR*  – 1)), where RR = *exp* (0.91 × *β*/*σ*_cIMT_). These results correspond to an e-value of 1.26 + sqrt (1.26 × (1.26 – 1) = 1.84 (1.15 for lower limit) for LDL-C and 2.00 (1.35 for lower limit) for nHDL-C. In the nHDL-C model, an unobserved confounder would need a strength of association of 2.00 on the risk ratio scale with both cIMT and high nHDL-C (≥130mg/dL) in order to fully account for the observed effect size.

Mean cIMT was not associated with TGs (β = 0.0144; *P* = 0.111) in either the non-fasting (β = 0.0122; *P* = 0.414) or fasting subgroups (β = -0.0132; *P* = 0.251) (**Table 3**), including after adjustment for other traditional CVD risk factors (non-fasting, β = 0.0150, *P* = 0.298; fasting, β = -0.0095, *P* = 0.385).

## DISCUSSION

In a cohort of adults in rural Uganda, we found significant relationships between carotid atherosclerosis and either LDL-C or nHDL-C, and no effect modification in these relationships by fasting status was observed. These findings support the feasibility of non-fasting blood collection for determination of CVD risk profiling in the region. To our knowledge, this is the first report investigating nHDL-C as a predictor of CVD risk in sSA, with potential implications for CVD risk assessment practices in resource-constrained settings where direct LDL-C measurement might be impractical, and fasting can be logistically difficult.

We found that nHDL-C and LDL-C levels were strongly and linearly correlated (β = 1.02; *r* = 0.922), as previously reported in Western Europe and North America [19]. Additionally, LDL-C and nHDL-C had nearly identical relationships with cIMT in both adjusted and unadjusted models. We did find that nHDL-C had nominally higher correlation with cIMT as compared to LDL-C, and thus could be a superior measure for risk prediction, although larger data sets and those that include clinical events are needed to validate this hypothesis. These relationships remained consistent in a sensitivity analysis restricted to directly measured LDL-C.

Although our results align with prior findings showing the utility of nHDL-C for CVD risk prediction in higher income settings [9], an unexpected result of our study was that LDL-C levels did not meaningfully differ by fasting status. We believe this was driven by low levels of TGs seen in both fasting and non-fasting groups (median TG = 114mg/dL, IQR = 91.5-153.5, and 130mg/dL, IQR = 95-178, respectively). Whether such an effect is due to genetic predisposition, dietary practices, level of physical activity, incidence of obesity and/or diabetes, or other characteristics of our rural sSA study population, is an important question for future studies. Prior work has also reported lower levels of TGs in rural African populations [51-53], suggesting that our data may be broadly generalizable to the region.

The magnitude of increase in cIMT as a function of higher nHDL-C and LDL-C in our study was similar to prior reports [44,54]. For example, we estimated an increase of approximately 0.08mm for each 100mg/dL increase in either lipid measure. Similar differences in cIMT – per 0.1mm increase – have been associated with increases of 10% to 15% in future risk of myocardial infarction and 13% to 18% for cerebrovascular accident [55]. Interestingly, in our study, this effect size diminished after adjusting for traditional CVD risk factors, but only in the fasting subgroup (**Table 3**). Because the decrease in effect size in adjusted models was seen for both LDL-C and nHDL-C, it is unlikely to be related to fasting status itself. Rather, we suspect this phenomenon was due to age as a confounding variable because age and lipids were correlated in the fasting subgroup (*P* = 0.012 for LDL-C and 0.001 for nHDL-C), but not the non-fasting subgroup (*P* = 0.697 for LDL-C and 0.660 for nHDL-C). When age was removed from our models, relationships between LDL-C, nHDL-C, and cIMT remained similar to unadjusted models of the fasting subgroup. Thus, we suspect that the differing relationships observed in the multivariable models were due to a cohort effect in our study, particularly the younger age in the fasting subgroup. To our knowledge, no prior study has found age to confound the relationship between cIMT and lipid levels.

We did not find a relationship between TGs and cIMT. There is little consensus in the existing literature about their relationship, although recent evidence has accumulated for TG as an independent predictor of future CVD risk. For example, the EPIC-Norfolk study found that elevated TGs are independently associated with increased risk of CVD, after adjustment for traditional risk factors [18]. The Health Professionals Follow-up Study also found a larger significant hazard ratio for future CVD for TGs compared to LDL-C [56]. We hypothesize that the lack of an association seen in our study is likely related to the low and narrow distribution of TGs in our study population. As such, this lack of correlation between cIMT and TGs helps establish the need for future studies to investigate if and how traditional Western European and North American CVD risk factors predict CVD outcomes in other populations globally.

We also did not observe effect modification by HIV serostatus on the association between lipids and cIMT in our adjusted models, even though the proportion of participants living with HIV was higher in the fasting subgroup. Notably, 100% of HIV-positive participants in the cohort were on ART, and 94% had virologic suppression. Only 7% participants were on protease inhibitors and none were on stavudine, which are the antiviral therapies most likely to induce hyperlipidemia and hypertriglyceridemia [57-60]. These results offer reassuring data that recommendations for non-nucleoside reverse transcriptase inhibitor-based first-line ART might be associated with relatively neutral lipid effects in sSA.

Our study is strengthened by a relatively large sample size, standardized laboratory methods, and a unique study population from rural sSA that includes older aged individuals and that is enriched for people living with HIV on antiretroviral therapy. The attributes of this study population make our study results applicable to other rural African communities who may share similar lifestyle, health care, and genetic factors. The use of cIMT as a surrogate marker for carotid atherosclerosis provides a valuable means of assessing CVD risk, but is not a substitute for larger studies that accrue CVD events to corroborate such findings. Another limitation of our study is the non-random assignment of fasting status. We attempted to mitigate the risk of confounding by use of regression models adjusted for CVD risk factors, including age, sex, smoking history, validated measures of diet and physical activity, and direct measurement of body anthropomorphics and blood pressure, which were largely similar between groups. In addition, e-value analysis suggests that unobserved confounding would need to be strong in order to account for estimated associations. However, we cannot exclude residual confounding as a possible cause of muted associations between lipids and cIMT in the fasting sub-group, and this relationship should be further explored in future studies. Lastly, the relatively small sample size in the non-fasting subgroup compared to our fasting subgroup limits our ability to make stronger conclusions about relationships between lipids and CVD risk in these subgroups.

To our knowledge, this is the first study to explore the utility of nHDL-C as a primary risk assessment tool for CVD in sSA. We observed that nHDL-C and LDL-C were similarly correlated with carotid atherosclerosis, and were not affected by fasting status. Because fasting lipid measurements pose a number of challenges in resource limited settings, our data support the use of nHDL-C for CVD risk stratification in the region. Widespread food and water insecurity [61,62] and the cultural insensitivity of unnecessary fasting in sSA and other low-income regions lends advantages to using non-fasting lipids in CVD risk assessment rather than following traditional guidelines established by studies conducted in Western European and North American countries. Future studies should evaluate relationships between lipids and CVD events, and examine similar relationships across diverse ethnic groups and among urban-dwelling populations.

## Additional material

Online Supplementary Document
