# Supplementary material for: To fast or not to fast: Lipid measurement and cardiovascular disease risk estimation in rural sub-Saharan Africa
Source: J Glob Health. 2020 Mar 17;10(1):010407. doi: 10.7189/jogh.10.010407 (PMC7101029; doi:10.7189/jogh.10.010407)
Supplement: Online Supplementary Document [file jogh-10-010407-s001.pdf]

Table S1. Sensitivity analysis, unadjusted and adjusted models for direct LDL-C (mg/dL) on carotid intima media thickness (mm), per 100-unit increase‡

Fasting Group	Unadjusted			Adjusted*		
	β coefficient (95% CI)	*P*-value	R-squared value	β coefficient (95% CI)	*P*-value	R-squared value
Non-fasting	0.0990 (-0.0304, 0.228)	0.129	0.068	0.119 (-0.0044, 0.2818)	0.144	0.494
Fasting	0.0760 (0.0218, 0.130)	0.006	0.044	0.0206 (-0.0264, 0.0677)	0.388	0.461
Total	0.0875 (0.0376, 0.137)	0.001	0.056	0.0457 (0.0015, 0.0898)	0.043	0.415

*Analysis conducted for 202 participants for whom direct LDL-C measurements were obtained.

Table S2. Effect Modification of Fasting Status and HIV Status on the Relationship Between cIMT and LDL-C, nHDL-C in Adjusted Models

	**Fasting Status**				**HIV Status**			
	**Interaction term β coeff. (95% CI)**	***P*-value**	**β coeff. (95% CI)**	***P*-value**	**Interaction term β coeff. (95% CI)**	***P*-value**	**β coeff. (95% CI)**	***P*-value**
**LDL-C level (mg/dL), per 100 unit increase**	-0.0112 (-0.0360, 0.0135)	0.372	0.0595 (0.0202, 0.0987)	0.003	0.0268 (-0.0387, 0.0923)	0.421	0.0341 (-0.0172, 0.0854)	0.191
**Non-HDL-C level (mg/dL), per 100 unit increase**	-0.00761 (-0.0265, 0.0112)	0.427	0.0476 (0.0133, 0.0820)	0.007	0.00789 (-0.0510, 0.0667)	0.792	0.0373 (-0.00872, 0.0833)	0.112

Table S3. Association between LDL-C, nHDL-C and Age, by Fasting Status

**Lipid Measure**	**Fasting Group**	**Age (per year increase)**		
		**β coefficient (95% CI)**	***P*-value**	**R-squared value**
**LDL-C level (mg/dL), per 100 unit increase**	Non-fasting	-1.02 (-6.22, 4.17)	0.697	0.002
	Fasting	3.94 (0.86, 7.02)	0.012	0.029
	Total	2.43 (-0.22, 5.09)	0.073	0.011
**Non-HDL-C level (mg/dL), per 100 unit increase**	Non-fasting	-1.00 (-5.53, 3.52)	0.660	0.002
	Fasting	4.80 (2.00, 7.59)	0.001	0.052
	Total	3.07 (0.67, 5.46)	0.012	0.021
